# The burden of attempted hanging and drowning presenting to hospitals in Ireland between 2007 and 2019: a national registry-based study

**DOI:** 10.1007/s00127-023-02525-w

**Published:** 2023-07-31

**Authors:** Philippa White, Paul Corcoran, Eve Griffin, Ella Arensman, Peter Barrett

**Affiliations:** 1 Department of Public Health (Cork & Kerry), HSE-South, St Finbarr’s Hospital, Douglas Road, Cork, Ireland; 2 National Suicide Research Foundation, 4 Western Gateway Building, Western Road, Mardyke, Cork, Ireland; 3 School of Public Health, University College Cork, 4th Floor, Western Gateway Building, Western Road, Cork, Ireland

**Keywords:** Deliberate self-harm, Attempted hanging, Attempted drowning, Suicide

## Abstract

**Purpose:**

To measure the impact of hospital-treated self-harm by hanging and drowning in Ireland in 2007–2019 and identify risk factors for these methods of self-harm.

**Method:**

Data on all self-harm presentations to Irish hospitals between 2007 and 2019 were obtained from the National Self-Harm Registry Ireland, a national self-harm surveillance system. Multinomial regression was used to explore factors associated with attempted hanging and drowning.

**Results:**

The age-standardised incidence rate of attempted hanging and drowning increased by 126% and 45%, respectively, between 2007 and 2019. The incidence of both methods was highest among young people aged 15–24 years. The odds of presenting to hospital for attempted hanging were highest in males (aOR 2.85, 95% CI 2.72–3.00), people experiencing homelessness (aOR 1.32, 95% CI 1.16–1.49) and individuals living in the capital, Dublin (aOR 1.23, 95% CI 1.17–1.29). The odds of presenting for attempted drowning were highest in males (aOR 1.68, 95% CI 1.58–1.78) and people experiencing homelessness (aOR 2.69, 95% CI 2.41–2.99).

**Conclusion:**

The incidence of hospital-treated self-harm by hanging and drowning is increasing in Ireland and is highest among adolescents and young adults. Males and people experiencing homelessness may be at highest risk and warrant targeted preventive interventions.

**Supplementary Information:**

The online version contains supplementary material available at 10.1007/s00127-023-02525-w.

## Introduction

Suicide is a major public health concern globally and the World Health Organization (WHO) estimates that worldwide, on average, one person dies from suicide every 40 s [1]. For every suicide, however, there are many more incidents of self-harm, and previous self-harm has been shown to be the strongest risk factor for suicide [1, 2]. For example, an estimated half of all individuals who die from suicide have previously self-harmed and the overall suicide rate among individuals who self-harm is estimated to be 100 times greater than the rate among the general population [3, 4]. Highly lethal methods of self-harm, such as attempted hanging and drowning, as well as other methods such as the use of firearms and jumping from a height, are methods that, by definition, have a high lethality and are more likely to lead to death by suicide than other self-harm methods [5]. These methods tend to be more violent, are generally rapidly lethal and reduce the possibility of detection, intervention or being able to change one’s mind [6, 7].

Attempted hanging and drowning are the two most common highly lethal methods of self-harm used in Ireland. The proportion of presentations to hospitals in Ireland for self-harm due to hanging was reported to be 3.8% in 2007, rising to 8.3% in 2019. This increase in the proportion of presentations for hanging was seen for both men and women, accounting for 6.2% and 1.9% of all presentations in men and women in 2007, respectively, rising to 12% and 5.2% of all presentations in men and women in 2019, respectively. The proportion of self-harm episodes due to drowning has increased more modestly over time, with 2.5% of all episodes involving attempted drowning in 2007, increasing to 3.9% of all episodes in 2019; an increase was observed in both men (3.3% in 2007 increasing to 5.1% in 2019) and women (1.9% in 2007, rising to 2.8% in 2019). This suggests an increasing burden of highly lethal methods of self-harm in the Irish population. Additionally, hanging and drowning are the most common methods of suicide in Ireland. In 2007–2012, hanging accounted for 74% and 60% of all deaths from suicide in men and women, respectively, and drowning accounted for 9% and 18% of all deaths in men and women, respectively [8]. Moreover, as methods of self-harm, attempted hanging and drowning are associated with a higher risk of death by suicide compared with many, if not most, other methods [9–12]. In spite of their high associated risk of death by suicide and their appreciable contribution to the burden of self-harm and suicide in Ireland, no study to date has analysed the trends and risk factors relating to attempted hanging and drowning in Ireland. It is essential to do so, however, in order to provide information for action to prevent self-harm and suicide by these highly lethal methods. Additionally, it is an important part of the wider efforts to attain the United Nations (UN) Sustainable Development Goal (SDG) Target 3.4 of reducing by one third premature mortality from non-communicable diseases through, among other things, the promotion of mental health and well-being [13].

The objectives of this study are to estimate the crude and age-standardised incidence rates of hospital-treated self-harm by hanging and drowning in Ireland between 2007 and 2019, to identify factors associated with hospital presentations for attempted hanging and drowning, and to identify factors associated with repeat hospital presentations for attempted hanging and drowning.

## Materials and methods

### Data sources

The National Self-Harm Registry Ireland (NSHRI), established by the National Suicide Research Foundation (NSRF) in 2000, is a national surveillance system of self-harm presentations to emergency departments (EDs) in Irish hospitals. The Registry aims to determine and monitor the incidence of hospital-treated self-harm in the Republic of Ireland, hereafter referred to as Ireland, and identify groups and areas in Ireland with a high incidence of self-harm. Data Registration Officers (DROs) collect data on self-harm presentations to the EDs of all acute hospitals in Ireland. For each self-harm presentation, the independently trained DROs collect and code a minimum dataset from individuals’ medical charts and hospital administrative records; these include sex, date of birth, method of self-harm used, and hour and date of attendance at the ED. A more detailed description of the NSHRI’s data collection and quality assurance procedures can be found elsewhere [14]. Data on all self-harm presentations to EDs in Ireland between 2007 and 2019 were obtained for this study.

Population data were obtained from the Central Statistics Office (CSO), Ireland's national statistical office. Data from the two most recent population censuses in 2011 and 2016 were used to provide population denominator data for those years and the CSO’s annual population estimates were used as denominators for intercensal years.

### Study population

The study population consisted of all individuals who presented to the EDs of all 33 acute hospitals in Ireland for treatment after an episode of self-harm between 1 January 2007 and 31 December 2019. There was no restriction by age.

### Variables

Variables to include in the regression models were selected a priori and the selection was made through an iterative, consensus-based process among the research team. Demographic characteristics, information about the episode of self-harm and information about the hospital presentation were captured by the Registry. For the purpose of this study, age was categorised into groups (i.e. < 15, 15–24, 25–34, 35–44, 45–54 and ≥ 55 years). Sex was coded as a binary variable (male/female); county of residence was also collapsed into a binary variable of area of residence (living in Dublin/living outside of Dublin). Residence type was determined by the location in which the individual was residing directly prior to presenting to hospital and was coded as private resident, homeless and other, the latter including prisoners and hospital inpatients, among others. Deprivation was captured by the Pobal Haase–Pratschke (HP) Deprivation Index, which was assigned to the electoral division in which the individual was living; the electoral division is the smallest legally-defined administrative area in the country and of which there are 3440 [15]. The Pobal HP Deprivation Index is a census-based, area deprivation index and is the main deprivation index used in Ireland [16]. The Pobal HP Deprivation Index for the electoral divisions of the individuals presenting were divided into quintiles and assigned numbers, with one representing the 20% most deprived areas and five representing the 20% most affluent areas.

Evidence of acute alcohol consumption at the time of self-harm or presentation to the ED, as denoted in the medical chart, was coded as a binary variable (yes/no). Both the time and day of presentation were coded as binary variables (in-hours, 9 am–5 pm/out-of-hours, 5 pm–9 am and weekday/weekend, respectively). The season of presentation was recoded from month and recorded as a categorical variable, as was the year of presentation.

The outcome variable was the method of self-harm recorded at time of presentation and was coded as hanging, drowning, and all other methods. Presentations in which the individual engaged in multiple self-harm methods concurrently were captured, as the Registry can record up to five self-harm methods for each presentation. The method of self-harm is recorded in the Registry according to the International Statistical Classification of Diseases and Related Health Problems (ICD-10) codes; self-harm by hanging is coded as ICD-10 X70 and self-harm by drowning as ICD-10 X71.

### Statistical analysis

Descriptive statistics were used to describe the presentations for attempted hanging and drowning. Counts and proportions were used to describe all categorical variables. Complete case analysis was used to manage missing data for variables in which < 5% of values were missing.

The crude annual incidence of presentations for attempted hanging and drowning per 100,000 population were calculated. Analyses were also performed for males, females, and all age groups separately. Direct standardisation, using the European Standard Population as the reference population, was employed to calculate the European age-standardised incidence rates (EASRs) of presentations for attempted hanging and drowning in 2007–2019 [17].

Multinomial regression was used to compare the odds of individuals having hospital presentations for attempted hanging, drowning, and all other self-harm methods, according to patient characteristics, aspects of the self-harm episode and variations in the time of presentation to the ED. A small number of individuals had hospital presentations for both hanging and drowning and it was decided a priori, for the purposes of multinomial regression, to classify these individuals as those who self-harmed by drowning to avoid any possible issues relating to data sparsity, as there were a smaller number of individuals who self-harmed by drowning than hanging. Logistic regression was used to explore factors associated with individuals who had any repeat hospital presentation for attempted hanging following this method as an index presentation, and a separate logistic regression model was used to explore factors associated with individuals who had any repeat hospital presentation for attempted drowning, following this method as an index presentation.

The deprivation index was only available for self-harm presentations occurring in the years 2015–2019. Consequently, sensitivity analyses including the deprivation index were conducted to assess the association between deprivation and attempted hanging and drowning, as well as repeat hanging and drowning attempts.

All *p* values reported were two-sided and *p* < 0.05 was considered statistically significant. Statistical analyses were performed using Stata version 17 (Statacorp, College Station, TX, USA).

## Results

There were a total of 152,489 presentations to EDs for self-harm in Ireland between 2007 and 2019, 9719 (6.4%) of which were presentations for attempted hanging and 4637 (3.0%) presentations for attempted drowning (Table 1). Men accounted for the majority of presentations for attempted hanging (68.3%) and drowning (59.0%); this is compared to 43.6% of presentations for all other methods of self-harm. Individuals younger than 35 years made up the majority of presentations for hanging (66.2%), drowning (55.1%), and all other self-harm methods (57.9%). Among women, those aged younger than 35 years accounted for 67% of presentations for attempted hanging, compared with 56% for attempted drowning and 58% for all other self-harm methods (Supplementary Table 1). In men, those aged younger than 35 years accounted for 66% of hanging presentations, compared with 54% for attempted drowning presentations and 58% for all other methods of self-harm. Most presentations for attempted hanging and drowning occurred out-of-hours (between 5 pm and 9 am), and on weekdays, but there was no clear seasonal pattern in the presentations.

**Table 1:** Characteristics of individuals at time of presentation to emergency departments after attempted hanging, drowning and other self-harm, Ireland, 2007–2019 (*n* = 152,489)

		Hanging presentations (*n* = 9,719) *n* (%)	Drowning presentations (*n* = 4,637) *n* (%)	Presentations for all other methods (*n* = 138,247) *n* (%)
Age (years)	< 15	357 (3.67)	28 (0.60)	4033 (2.92)
	15–24	3304 (33.99)	1234 (26.61)	43,195 (31.24)
	25–34	2768 (28.48)	1295 (27.93)	32,829 (23.75)
	35–44	1784 (18.36)	888 (19.15)	27,652 (20.00)
	45–54	995 (10.24)	691 (14.90)	19,121 (13.83)
	≥ 55	511 (5.26)	501 (10.80)	11,417 (8.26)
Sex	Male	6638 (68.30)	2737 (59.03)	60,266 (43.59)
	Female	3081 (31.70)	1900 (40.97)	77,981 (56.41)
Area of residence	Dublin	3611 (37.15)	1176 (25.36)	45,965 (33.25)
	Outside of Dublin	6108 (62.85)	3461 (74.64)	92,282 (66.75)
Residence type	Private resident	8884 (91.47)	4048 (87.30)	128,137 (92.70)
	Homeless	433 (4.46)	432 (9.32)	5225 (3.78)
	Other	395 (4.07)	157 (3.39)	4,868 (3.52)
Alcohol consumed	Yes	2870 (29.53)	2089 (45.05)	49,855 (36.06)
	No	6849 (70.47)	2548 (54.95)	88,392 (63.94)
Time of	9 am–5 pm	3038 (31.26)	1138 (24.59)	40,125 (29.14)
presentation	5 pm–9 am	6635 (68.27)	3489 (75.41)	97,565 (70.86)
Day of	Weekday	6927 (71.27)	3123 (67.35)	97,399 (70.45)
presentation	Weekend	2792 (28.73)	1514 (32.65)	40,848 (29.55)
Season of	Spring	2361 (24.29)	986 (21.26)	34,327 (24.83)
presentation	Summer	2622 (26.98)	1282 (27.65)	35,748 (25.86)
	Autumn	2467 (25.38)	1306 (28.16)	34,987 (25.31)
	Winter	2269 (23.35)	1063 (22.92)	33,185 (24.00)

Note: Some individuals presented more than once for attempted hanging, drowning and all other methods

### Attempted hanging

**Fig. 1:**
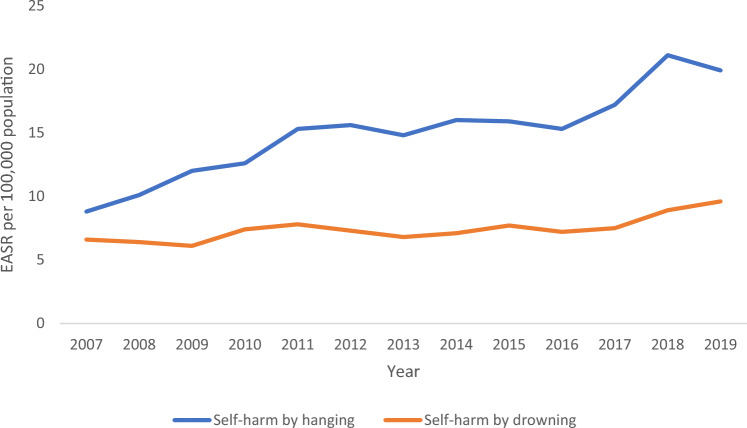
European age-standardised incidence rates (EASRs) of hospital presentations for attempted hanging and drowning per 100,000 population, Ireland, 2007–2019

The crude incidence rate of presentations for attempted hanging more than doubled during 2007–2019, rising from 10.2 to 20.9 presentations per 100,000 population. Similarly, the EASR of presentations for attempted hanging increased by 126% between 2007 and 2019, rising from 8.8 to 19.9 presentations per 100,000 population (Fig. 1). The incidence, both crude and EASR, was consistently highest in the 15–24 years age group, followed by the 25–34 years age group, and was consistently higher in men than women.

9719 hospital presentations for attempted hanging in Ireland in 2007–2019 were made by 8170 individuals. Compared with other self-harm methods, the odds of presenting to hospital for attempted hanging were highest in males (aOR 2.85, 95% CI 2.72–3.00), people experiencing homelessness (aOR 1.32, 95% CI 1.16–1.49) and those living in the capital, Dublin (aOR 1.23, 95% CI 1.17–1.29) (Table 2).

**Table 2:** Multinomial regression model of risk and protective factors associated with individuals presenting for attempted hanging and drowning compared with all other self-harm methods, Ireland, 2007–2019 (*n* = 95,114)

		Attempted hanging aOR (95% CI)	Attempted drowning aOR (95% CI)
Age group (years)	< 15	1.22 (1.08, 1.38)	0.31 (0.21, 0.45)
	15–24	1.00 (ref.)	1.00 (ref.)
	25–34	1.15 (1.08, 1.22)	1.31 (1.20, 1.43)
	35–44	0.96 (0.90, 1.03)	1.14 (1.04, 1.26)
	45–54	0.82 (0.75, 0.89)	1.35 (1.22, 1.50)
	≥ 55	0.59 (0.53, 0.66)	1.46 (1.30, 1.64)
Sex	Female	1.00 (ref.)	1.00 (ref.)
	Male	2.85 (2.72, 3.00)	1.65 (1.55, 1.77)
Area of residence	Dublin	1.23 (1.17, 1.29)	0.67 (0.62, 0.72)
	Outside Dublin	1.00 (ref.)	1.00 (ref.)
Residence type	Private resident	1.00 (ref.)	1.00 (ref.)
	Homeless	1.32 (1.16, 1.49)	3.98 (3.51, 4.52)
	Other	1.22 (1.08, 1.38)	1.29 (1.08, 1.54)
Alcohol consumed	Yes	0.74 (0.70, 0.78)	1.33 (1.25, 1.43)
	No	1.00 (ref.)	1.00 (ref.)
Time of presentation	In-hours (9 am–5 pm)	1.08 (1.03, 1.14)	0.83 (0.77, 0.90)
	Out-of-hours (5 pm–9 am)	1.00 (ref.)	1.00 (ref.)
Day of presentation	Weekday	1.00 (ref.)	1.00 (ref.)
	Weekend	0.96 (0.91, 1.01)	1.13 (1.06, 1.21)
Season of	Spring	1.00 (ref.)	1.00 (ref.)
presentation	Summer	1.11 (1.04, 1.18)	1.22 (1.11, 1.34)
	Autumn	1.04 (0.98, 1.11)	1.32 (1.20, 1.44)
	Winter	0.98 (0.92, 1.05)	1.12 (1.02, 1.24)

*CI*, confidence interval, *OR*, odds ratio, *ref*, reference category

Of the 8170 individuals who had hospital presentations for attempted hanging, 994 (12.2%) had at least one repeat presentation for attempted hanging during the study period. The odds of having a repeat presentation for attempted hanging were highest in individuals not living in a private household (people experiencing homelessness, aOR 2.15, 95% CI 1.63–2.83, and those residing in ‘other’ settings, aOR 1.53, 95% CI 1.13–2.09) and individuals living in the capital, Dublin (aOR 1.31, 95% CI 1.14–1.50) (Table 3). Conversely, the odds of having a repeat presentation for attempted hanging were lowest in males (aOR 0.82, 95% CI 0.72–0.95) and older adults aged ≥ 55 (aOR 0.65, 95% CI 0.46–0.94).

**Table 3:** Logistic regression model of risk and protective factors associated with repeat presentations for attempted hanging (*n* = 8,123)

		aOR (95% CI)
Age group (years)	< 15	0.77 (0.51, 1.16)
	15–24	1.00 (ref.)
	25–34	1.10 (0.93, 1.31)
	35–44	1.10 (0.91, 1.33)
	45–54	1.07 (0.84, 1.35)
	≥ 55	0.65 (0.46, 0.94)
Sex	Female	1.00 (ref.)
	Male	0.82 (0.72, 0.95)
Area of residence	Dublin	1.31 (1.14, 1.50)
	Outside Dublin	1.00 (ref.)
Residence type	Private resident	1.00 (ref.)
	Homeless	2.15 (1.63, 2.83)
	Other	1.53 (1.13, 2.09)
Alcohol consumed	Yes	1.09 (0.94, 1.26)
	No	1.00 (ref.)
Time of presentation	In-hours (9 am–5 pm)	0.74 (0.63, 0.86)
	Out-of-hours (5 pm–9 am)	1.00 (ref.)
Day of presentation	Weekday	1.00 (ref.)
	Weekend	1.05 (0.90, 1.21)
Season of presentation	Spring	1.00 (ref.)
	Summer	1.09 (0.90, 1.31)
	Autumn	1.04 (0.86, 1.26)
	Winter	1.11 (0.92, 1.35)

*aOR*, adjusted odds ratio, *CI*, confidence interval, *ref*, reference category

### Attempted drowning

The crude incidence rate of presentations for attempted drowning rose during 2007–2019 from 7.2 presentations per 100,000 population in 2007 to 9.8 in 2019. The EASR also increased by 45%, rising from 6.6 presentations per 100,000 population in 2007 to 9.6 presentations per 100,000 population in 2019 (Fig. 1). The crude incidence rate and EASR of presentations for attempted drowning were highest in the 15–24 years and 25–34 years age groups and were higher in men than women.

The 4637 hospital presentations for attempted drowning in Ireland in 2007–2019 involved 4,010 individuals. Compared with other self-harm methods, the odds of presenting to hospital for attempted drowning were highest in males (aOR 1.65, 95% CI 1.55–1.77) and people experiencing homelessness (aOR 3.98, 95% CI 3.51–4.52) (Table 2).

Of the 4,010 individuals who had hospital presentations for attempted drowning, 414 (10.3%) had more than one hospital presentation for attempted drowning presentation. People experiencing homelessness (aOR 2.52, 95% CI 1.86–3.41) had the highest odds of a repeat presentation for attempted drowning (Table 4).

**Table 4:** Logistic regression model of risk and protective factors associated with repeat presentations for attempted drowning (*n* = 3973)

		aOR (95% CI)
Age group (years)	< 15	–
	15–24	1.00 (ref.)
	25–34	1.75 (1.31, 2.34)
	35–44	1.35 (0.97, 1.87)
	45–54	1.52 (1.08, 2.14)
	≥ 55	1.50 (1.02, 2.21)
Sex	Female	1.00 (ref.)
	Male	0.88 (0.71, 1.08)
Area of residence	Dublin	1.09 (0.86, 1.38)
	Outside Dublin	1.00 (ref.)
Residence type	Private resident	1.00 (ref.)
	Homeless	2.52 (1.86, 3.41)
	Other	1.28 (0.75, 2.20)
Alcohol consumed	Yes	1.17 (0.95, 1.44)
	No	1.00 (ref.)
Time of presentation	In-hours (9 am–5 pm)	0.79 (0.61, 1.02)
	Out-of-hours (5 pm–9 am)	1.00 (ref.)
Day of presentation	Weekday	1.00 (ref.)
	Weekend	1.00 (0.81, 1.25)
Season of presentation	Spring	1.00 (ref.)
	Summer	0.85 (0.63, 1.13)
	Autumn	0.88 (0.66, 1.16)
	Winter	0.76 (0.56, 1.04)

*aOR*, adjusted odds ratio, *CI*, confidence interval, *ref*, reference category

### Sensitivity analyses

Inclusion of the deprivation variable into the analyses demonstrated that individuals living in the most deprived areas had the highest odds of hospital presentations for both attempted hanging (vs. individuals living in the least deprived areas, aOR 2.26, 95% CI 1.49–3.44) and drowning (aOR 3.47, 95% CI 2.25–5.36). Similarly, individuals living in the most deprived areas had the highest odds of presenting to hospital for repeated hanging attempts (vs. individuals living in the least deprived areas, aOR 4.55, 95% CI 1.49–13.91) and repeated drowning attempts, although the latter did not reach statistical significance (aOR 2.64, 95% CI 0.74–9.42). The residence type variable was omitted from the regression models in the sensitivity analyses due to a high level of collinearity between it and the deprivation variable.

## Discussion

### Summary of main findings

This study has shown that the incidence of presentations for attempted hanging more than doubled between 2007 and 2019, while the incidence of presentations for attempted drowning increased by almost 50%. Notably, the incidence of presentations for both methods was highest in young people aged 15–24 years and in males. Males, people experiencing homelessness and those living in the capital city, Dublin, had the highest odds of presenting for attempted hanging, compared with presenting for all other self-harm methods. However, women, non-private household residents and those living in the capital were more likely to present with repeated episodes of attempted hanging, following an index presentation to hospital. Males and people experiencing homelessness had the highest odds of presenting for attempted drowning, while people experiencing homelessness had the highest odds of a repeated episode of attempted drowning following an index presentation to hospital.

### Comparison of findings with existing literature

The findings of this study were largely consistent with the existing international literature. Firstly, males had higher odds than females of presenting to hospital after attempted hanging and attempted drowning. In studies from Europe, the USA and Australia, men have been found to be at greater risk of suicide by hanging than women [7, 18, 19]. This finding has also been observed in older adults, and after adjusting for confounding factors [18, 19]. Similarly, a systematic review of the global literature on suicide and self-harm by drowning observed that there were a small preponderance of males engaging in suicide by drowning, which is consistent with the findings of this study [20].

An almost inverse relationship was observed between age and odds of hospital presentation for attempted hanging. This is in line with previous Irish and international studies which reported that victims of hanging suicides were younger than victims of suicide from other methods, and that younger age groups were at higher risk of intentional hanging than older age groups [21, 22].

Similarly, studies from Europe and Australia indicate that older adults may have the highest risk of suicidal drowning compared with other age groups, and this study reported the same finding in relation to age and self-harm by drowning [23, 24].

In keeping with the existing literature, this study found that area deprivation was associated with self-harm by hanging and drowning, with individuals living in the most deprived areas having the highest odds of presenting to hospital after an episode of attempted hanging or drowning. Observational studies conducted in Canada and the United States (US) have found similar positive associations between higher levels of deprivation and risk of suicide by hanging in both males and females and among adolescents [25, 26]. No studies were identified, however, which explored the relationship between deprivation and risk of suicide or self-harm by drowning.

Correspondingly, this study found that individuals experiencing homelessness had considerably higher odds of attempted hanging and drowning, as well as repeated attempts, than individuals living in a private residence or other setting. This is largely consistent with another study performed in Ireland that observed a distinctly higher incidence of all methods of self-harm among homeless individuals compared with individuals with a fixed residence [27]. Moreover, a study of people experiencing homelessness presenting to three EDs in the United Kingdom (UK) found that homeless individuals had a higher risk of self-harm repetition than domiciled individuals [28].

### Strengths and limitations

This study had a number of strengths. Firstly, this study availed of data from the NSHRI, one of the few national self-harm surveillance systems worldwide [1]. Several countries have self-harm surveillance systems, however, many lack national coverage, or only collect information on self-harm that results in admission to an inpatient hospital ward [29]. Secondly, the NSHRI has had complete coverage of the EDs in all acute hospitals in Ireland since 2006, which afforded this study a comprehensive and national perspective of attempted hanging and drowning in Ireland [30].

Thirdly, the quality of the data collected by the NSHRI and analysed in this study was high. The NSHRI’s DROs are independently trained and apply the self-harm case definition and inclusion/exclusion criteria in a standardised, systematic manner. There are numerous quality control measures in place to ensure the validity of the data, including continual checks for consistency and accuracy and regular measurements of interrater reliability between DROs, which are high (e.g. Kappa statistic of 0.90 in 2017). Finally, while there is extensive evidence available about the factors associated with death by suicide by hanging and drowning, there is limited evidence on the factors associated with self-harm by hanging and drowning. This study provides valuable insights into the characteristics of individuals who present to hospital after highly lethal self-harm methods.

This study also had a number of limitations. Firstly, the NSHRI is a routine data source which collects a limited number of variables. The registry does not collect information on marital status, ethnicity, or history of mental illness in the individuals presenting to EDs after self-harm. These and other unmeasured variables may contribute towards the risk of attempted hanging and drowning and without their inclusion, there may have been residual confounding of the associations observed in this study [18, 31, 32]. Due to a number of factors, including the lack of an individual health identifier in Ireland, limited and inconsistent use of diverse electronic health record systems across all levels of the health system, and general practitioners largely existing as private practitioners who are not paid by the public health service, it is unfeasible to link the NSHRI data to other patient records to capture these potential confounders. Secondly, the Registry does not record specific details about the level of lethality of acts of attempted hanging or drowning. Thirdly, the NSHRI only collects information about self-harm, and not death by suicide. Deaths by suicide are recorded by the CSO in Ireland and these data are not linked to the NSHRI [33]. Therefore, the risk of death by suicide among those who self-harm by hanging and drowning cannot be readily calculated, nor can the case fatality rate of different methods of self-harm. It is possible that some of the individuals included in this dataset died by suicide during the study period and this may have biased the analyses assessing risk of repeated self-harm by hanging and drowning. For example, in this study, men were found to have lower odds of repeat hospital presentations for attempted hanging and drowning compared with women; while it is possible women were more likely than men to engage in repeated self-harm attempts, it is also possible that men were as, if not more, likely than women to engage in repeated episodes of self-harm by hanging and drowning, but their episodes of self-harm ultimately resulted in death by suicide, which were not captured by this study [31, 34].

Fourthly, the quality of data collected on acute alcohol consumption in self-harm presentations is less than it is for other variables in the NSHRI. All other variables are routinely recorded in the patient notes or in hospital administrative records. Acute alcohol consumption is not systematically recorded in the ED patient notes and there may be misclassification of acute alcohol consumption status. Therefore, associations between acute alcohol consumption and attempted hanging and drowning should be interpreted with caution. Furthermore, while the NSHRI has complete coverage of all 33 acute hospitals in Ireland, attempted hanging and drowning incidents treated in primary care or on psychiatric inpatient wards, or which are untreated by the health service, are not captured by the NSHRI. This may underestimate the incidence of self-harm by hanging and drowning, or may bias the findings of the analyses exploring the factors associated with these methods of self-harm. Finally, the NSHRI does not collect information on where self-harm acts take place. This information could be very useful, as it could reveal public locations where intentional hanging or drowning repeatedly occur and these sites could be targeted for interventions to prevent suicide.

### Implications

Firstly, this study demonstrates that the rate of hospital presentations for both hanging and drowning increased steadily between 2007 and 2019. This occurred against a backdrop of fluctuating rates of hospital presentations for all methods of self-harm, suggesting that attempted hanging and attempted drowning comprise a growing problem compared with all other self-harm methods. Secondly, certain subsets of the population may be at increased risk of self-harm by hanging and drowning and these findings may help to inform self-harm and suicide prevention strategies. As adolescents and young adults may be at greatest risk of self-harm by hanging compared with other age groups, primary prevention of suicidal behaviour through school-based interventions that raise awareness about suicide or increase connectedness among students may be effective at addressing some of the risk factors for self-harm [35, 36]. Similarly, as homeless individuals appear to be at particularly high risk of intentional hanging and drowning and repetition of self-harm by these means, this population may need to be prioritised for effective therapeutic interventions for mental health conditions both before and after episodes of self-harm. Finally, previous research has shown that self-harm by hanging and drowning are associated with a higher risk of suicide than many other self-harm methods [9]; consequently, individuals who present to hospital after engaging in these highly lethal methods of self-harm should receive appropriate care, including a specialist, biopsychosocial assessment to identify the drivers of distress leading to self-harm, and follow-up care with inpatient or outpatient mental health services [37].

Self-harm and suicide by hanging and drowning are uniquely challenging methods of self-harm to prevent. Both methods can be rapidly lethal, reducing the probability of a life-saving intervention, and the means for carrying out either method are almost universally available [31, 34]. Additionally, compared with other self-harm methods, individuals who engage in these two highly lethal methods of self-harm already have a higher risk of death by suicide [9, 38]. Therefore, to prevent self-harm and suicide by hanging and drowning, the more distal determinants of self-harm and suicide, such as substance abuse, poor mental health, and a reduced sense of connectedness, must be addressed [39, 40]. Additionally, the impact of exposure to harmful media coverage of suicide and self-harm must be addressed. Sensationalist, detailed and extensive reporting of suicide in the media is associated with increased suicide and exposure to this harmful coverage can contribute to suicide contagion and copycat suicides, especially among vulnerable populations, such as young adults [41, 42]. Conversely, responsible media coverage that emphasises how individuals can overcome suicidal ideation and address mental health issues may encourage vulnerable individuals to seek help (“the Papageno effect”) [43]. Therefore, efforts must be made to ensure that not only media depictions of suicide are in accordance with media guidelines for reporting suicide and social media platforms rigorously enforce their policies on content related to suicide, but that media reporting of suicides has a positive preventive effect where possible.

As mentioned previously, hanging and drowning are uniquely difficult methods of self-harm to prevent. As most hanging suicides likely occur in private settings [31, 44], intervention by others is challenging. However, self-harm by drowning may occur in more public settings, where a limited number of interventions can be implemented acutely during incidents of self-harm. Voluntary organisations which provide suicide prevention patrols and monitor major waterways can be established to identify and provide assistance to individuals attempting to self-harm. However, strategies to avoid the presence of these organisations leading to greater public awareness about locations or ‘hotspots’ where intentional drowning repeatedly occurs would need to be considered. For example, closed-circuit television (CCTV) surveillance systems could allow volunteers to inconspicuously monitor a location and improving the lighting around waterways where intentional drowning occurs may be useful for not only discouraging individuals from self-harming there, but also increasing their chances of detection and intervention [45]. Finally, consideration can be given to erecting signposts which provide a contact number for a crisis helpline, or installing emergency telephones that connect the caller directly with a crisis helpline, at locations where intentional drowning is known to occur repeatedly [45].

## Conclusion

Self-harm by attempted hanging and drowning are growing public health problems in Ireland. Multiple subsets of the population are at risk of attempted hanging and drowning and, therefore, prevention strategies must be multifaceted and address proximal and distal risk factors across the life span. As the individuals who self-harm by hanging and drowning may be at the highest risk of completing suicide, addressing the many determinants of these highly lethal methods of self-harm should be a priority for suicide prevention strategies.

### Supplementary Information

Below is the link to the electronic supplementary material.

Supplementary file1 (PDF 94 KB)

## Data Availability

Data are not publicly available due their highly sensitive nature and their containing information that could compromise the privacy of individuals captured by the Registry. Extensive amounts of aggregate data are published in the NSRF’s annual reports.
