# Supplementary material for: The burden of attempted hanging and drowning presenting to hospitals in Ireland between 2007 and 2019: a national registry-based study
Source: Soc Psychiatry Psychiatr Epidemiol. 2023 Jul 31;59(2):235–44. doi: 10.1007/s00127-023-02525-w (PMC10838814; doi:10.1007/s00127-023-02525-w)
Supplement: Supplementary file 1 — Supplementary file1 (PDF 94 KB) [file 127_2023_2525_MOESM1_ESM.pdf]

Supplementary Information

Table S1. Gender-age groups of individuals at time of presentation to emergency departments after attempted hanging and drowning, Ireland, 2007-2019 (n=152,489)

		Hanging presentations (n=9,605) n (%)	Drowning presentations (n=4,637) n (%)	Presentations for all other methods (n=138,247) n (%)
Both genders				
Age (years)	<15	356 (3.71)	28 (0.60)	4,033 (2.92)
	15-24	3,265 (33.99)	1,234 (26.61)	43,195 (31.24)
	25-34	2,737 (28.50)	1,295 (27.93)	32,829 (23.75)
	35-44	1,765 (18.38)	888 (19.15)	27,652 (20.00)
	45-54	975 (10.15)	691 (14.90)	19,121 (13.83)
	≥55	507 (5.28)	501 (10.80)	11,417 (8.26)
Males				
Age (years)	<15	204 (3.10)	15 (0.55)	872 (1.45)
	15-24	2,198 (33.44)	684 (24.99)	17,871 (29.65)
	25-34	1,912 (29.09)	785 (28.68)	16,397 (27.21)
	35-44	1,240 (18.87)	558 (20.39)	12,696 (21.07)
	45-54	662 (10.07)	412 (15.05)	7,745 (12.85)
	≥55	356 (5.42)	283 (10.34)	4,685 (7.77)
Females				
Age (years)	<15	152 (5.01)	13 (0.68)	3,161 (4.05)
	15-24	1,067 (35.18)	550 (28.95)	25,324 (32.47)
	25-34	825 (27.20)	510 (26.84)	16,432 (21.07)
	35-44	525 (17.31)	330 (17.37)	14,956 (19.18)
	45-54	313 (10.32)	279 (14.68)	11,376 (14.59)
	≥55	151 (4.98)	218 (11.47)	6,732 (8.63)

Note: Some individuals presented more than once for attempted hanging, drowning and all other methods.
